# Nutritional profiling of breast muscle: A comparative study between Yuzhong pigeons and European meat pigeons

**DOI:** 10.1016/j.fochx.2025.102157

**Published:** 2025-01-04

**Authors:** Pengkun Yang, Xinghui Song, Wenyi Wu, Liheng Zhang, Zhanbing Han, Xinlei Wang, Runzhi Wang, Mingjun Yang, Zhen Zhang

**Affiliations:** a College of Animal Science and Technology, Henan University of Animal Husbandry and Economy, Zhengzhou 450046, China; b Nanjing Institute of Animal Husbandry and Poultry Science, Nanjing 210036, China; c Henan Tiancheng Pigeon Industry Co., Ltd, Pingdingshan 462500, China

**Keywords:** Pigeon, Nutrient composition, Amino acids, Fatty acids, Trace elements

## Abstract

This study compares the nutritional profiles of Yuzhong and European meat pigeons to inform breeding and consumer choices. Thirty 28-day-old pigeons were analyzed for protein, fat, moisture, amino acids, fatty acids, and trace elements in breast muscle samples. No significant differences were found in protein, fat, moisture, or essential amino acid levels between breeds. However, Yuzhong pigeons showed higher levels of umami amino acids, monounsaturated fatty acids (particularly oleic acid), and trace elements (copper, iron, zinc, selenium). In contrast, European meat pigeons were richer in polyunsaturated fatty acids, such as linoleic acid, arachidonic acid, and DHA. These results highlight nutritional distinctions between the breeds, providing guidance for breeding programs and offering insights for consumers seeking specific dietary attributes in poultry meat.

## Highlights

• Yuzhong pigeons are rich in umami amino acids and monounsaturated fats.
• European meat pigeons contain higher levels of polyunsaturated fatty acids.
• Yuzhong pigeons have elevated levels of copper, iron, zinc, and selenium.
• Nutritional profiles support diverse breeding and consumer dietary choices.
• Findings aid in enhancing pigeon meat as a health-conscious protein source.

## 1 Introduction

Poultry meat is an important source of high-quality protein, contributing significantly to human nutrition. Among various poultry products, pigeon meat stands out as a premium offering due to its rich nutritional profile and unique taste (Chen, 2019). With rising living standards and increasing demand for premium food products, pigeon meat has gained recognition beyond its traditional role as a protein source. It is distinguished by its rich nutritional profile, featuring low cholesterol levels and high protein content, making it a valuable component of a balanced human diet (Pomianowski et al., 2009). The pigeon meat industry, as a rising force within the specialty poultry sector, is increasingly recognized for its role in supplying high-end poultry products to meet the growing consumer demand for healthier and more diversified protein sources (Jin et al., 2023). The inclusion of pigeons in the official poultry category by Document No. 1 of the National Livestock Commission [2021] underscores their importance in modern animal husbandry. As part of the industry's restructuring, the pigeon meat sector has played a pivotal role in boosting farmers' income and supporting sustainable agricultural development.

China, as a global leader in both the production and consumption of pigeon meat, reported over 43.867 million breeding pairs and 584 million young pigeons in 2022 alone, reflecting the scale of the industry. In addition to China, other major producers of pigeon meat include the USA and Canada, with the USA alone producing approximately 2.5 million squabs annually (Jiang et al., 2019). In Europe, Great Britain, Italy, and France lead in pigeon meat production, while Hungary, Germany, Denmark, and Spain also contribute to its consumption (Cordero et al., 2019; Dal Bosco et al., 2005; Miąsko & Łukasiewicz, 2016). Pigeon meat holds a unique position in global nutrition, valued for its high protein content, low fat, and rich array of essential amino acids, vitamins, and minerals. It is widely regarded as a delicacy in many cultures, with significant culinary and nutritional importance extending to Europe, the Middle East, Africa, and parts of North America. The demand for pigeon meat continues to rise year by year, driven by the public's increasing interest in nutrition, health, and quality of life (Cai et al., 2022). Pigeon meat is prized for its excellent protein content, low fat levels, and rich amino acid composition, making it an ideal choice for health-conscious consumers seeking nutrient-dense alternatives to traditional meats. In China, the majority of the meat pigeons are imported breeds, with prominent varieties such as White King, European meat pigeons, Tyson pigeons, and Silver King pigeons dominating the market (Yuan et al., 2023). Among these, European meat pigeons are particularly favored for their superior meat production and well-developed chest muscles, which contribute to higher yields and greater commercial value (Li et al., 2023). A comparative study on the nutritional value of four Chinese local meat pigeon breeds and White King pigeons revealed that local breeds exhibited darker flesh, superior water retention, higher protein and inosine contents, a greater proportion of essential amino acids, and a lower saturated fatty acid ratio compared to White King pigeons (Chang, Tang, et al., 2023; Chang, Zhang, et al., 2023).

On the other hand, the Yuzhong pigeon, a local breed domesticated in Wugang, holds a special place in the domestic market. Though smaller in size, it is distinguished by its unique flavor and adaptability, qualities that make it a valuable genetic resource (Wang et al., 2023). The Yuzhong pigeon was officially listed as a key indigenous breed during the third National Survey of Livestock and Poultry Genetic Resources in 2023, reaffirming its significance in the national strategy for conserving local breeds (Zhang et al., 2023). Nutritionally, pigeon meat offers numerous benefits compared to other poultry. It is rich in essential amino acids, vitamins, and minerals, which are vital for human health, particularly for enhancing immune function, promoting muscle growth, and supporting overall metabolic health (Jin et al., 2023). As consumers become more health-conscious, pigeon meat's nutritional benefits position it as an attractive option for those seeking healthier, leaner protein sources.

The nutritional analysis of Yuzhong pigeons and European meat pigeons highlights their important contributions to human nutrition, particularly in terms of high-quality protein, essential amino acids, and low-fat content. European meat pigeons, known for their well-developed chest muscles, provide excellent meat yields with a high protein-to-fat ratio, making them particularly attractive to health-conscious consumers. In contrast, Yuzhong pigeons, while smaller in size, offer a unique flavor and a nutrient profile rich in vitamins and minerals essential for immune support and muscle development. By comparing the breast muscle composition of these two breeds, valuable insights can be gained for breeding programs aimed at optimizing the nutritional value of pigeon meat.

Pigeon meat is highly valued for its high-quality protein, essential amino acids, and beneficial fatty acids. Previous studies have analyzed its chemical composition, with Pomianowski et al. focusing on protein and fat content across breeds, and Chang et al. examining the amino acid and fatty acid profiles of European meat pigeons (Chang et al., 2017; Pomianowski et al., 2009). Research on local breeds, such as the Wugang pigeon, has highlighted their unique flavor and nutritional benefits. However, few studies have compared indigenous and imported breeds or explored trace elements and antioxidant properties, leaving gaps in understanding pigeon meat's full nutritional potential. This study addresses these gaps by comparing the amino acid, fatty acid, and trace element profiles of Yuzhong pigeons and European meat pigeons. The findings reveal unique advantages: Yuzhong pigeons have higher levels of monounsaturated fatty acids and trace elements, while European meat pigeons are richer in polyunsaturated fatty acids. These insights provide valuable guidance for breeding strategies and underscore the health benefits of pigeon meat, contributing to sustainable poultry production. We selected 28-day-old squabs for this study, as they represent the predominant age group in commercial squab production due to their optimal size, weight, and market value at the time of harvest. At this commercially relevant stage, the nutritional profiles of Yuzhong and European meat pigeons were analyzed and compared to identify their respective advantages.

## 2 Materials and methods

### 2.1 Experimental animals

A total of 60 pigeons, each aged 28 days, were selected for slaughter—30 European meat pigeons (0.53 ± 0.057 kg) and 30 Yuzhong pigeons (0.38 ± 0.035 kg). The experimental squabs were supplied by Henan Tiancheng Pigeon Industry Co., Ltd.

### 2.2 Feeding management

All experimental squabs were artificially incubated and reared using a 2 + 3 parent pigeon feeding system. The composition and nutrient levels of the parent pigeons' diets are presented in Table 1. The environmental conditions for the pigeons were maintained at 30 °C with 45 % humidity and natural ventilation. Daily management followed the guidelines outlined in the Manual of Breeding and Management of Meat Pigeons provided by Henan Tiancheng Pigeon Industry Co., Ltd..

**Table 1:** Composition and nutrient levels of the diets.

Item	Content (%)	Percentage (%)
Composition of granulated meal		50.00
Corn	40.88	
Soybean meal	24.90	
Fermented soybean	8.00	
Wheat	10.00	
Soybean oil	6.86	
Lysine	0.43	
Methionine	0.78	
Threonine	0.15	
Premix a	8.00	
Total	100.00	
Composition of whole-grain form feed		50.00
Corn	50.00	
Pea	50.00	
Total	100.00	
Calculated nutrient levels		
Metabolizable energy (Mcal/kg)	2.97	
Crude protein	17.29	
Calcium	0.95	
Available phosphorus	0.38	
Lysine	1.10	
Methionine + Cysteine	0.76	

a Premix provided the following per kilogram of diet: vitamin A, 8000 U; vitamin D3, 300 IU; vitamin E, 40 mg; vitamin K3, 1.6 mg; vitamin Bl, 5 mg; vitamin B2, 22 mg; vitamiB6, 4 mg; vitamin B12, 50 μg; niacin, 30 mg; folic acid, 0.6 mg; pantothenic acid, 6.4 mg; biotin, 0.2 mg; sodium chloride, 760 mg; copper, 24 mg; iron, 76 mg; zinc, 80 mg; manganese, 116 mg; selenium, 0.4 mg.

### 2.3 Sampling

**Fig. 1:**
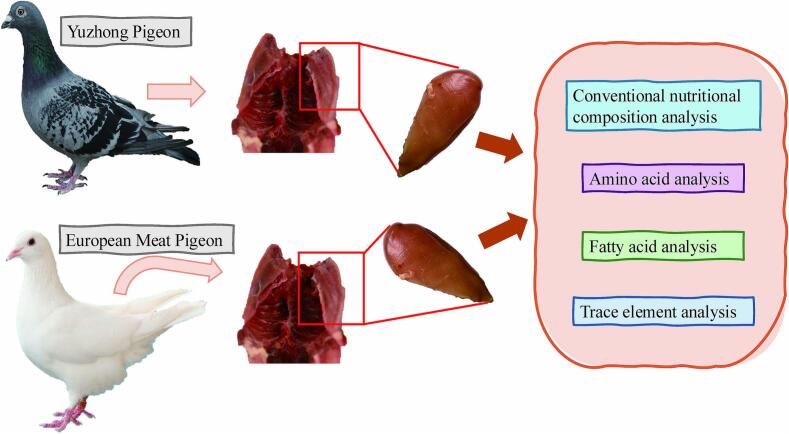
The experimental workflow, outlining the comparative analysis of the breast muscle's conventional nutritional composition, including amino acids, fatty acids, and trace elements, in both Yuzhong pigeons and European meat pigeons.

All experiments in this study were conducted in accordance with a protocol approved by the Institutional Animal Care and Use Committee (IACUC) in China, under ethical approval code HNUAHEER 23104 (20th April. 2023). The pigeons were fasted for 12 h the night before slaughter, with feed withheld starting the previous evening. At 8:00 AM the next day, researchers at Henan College of Animal Husbandry and Economics anesthetized pigeons with 10 mg/mL ketamine/xylazine, followed by bloodletting and cervical dislocation. The right breast muscles were stored at −20 °C for subsequent nutritional analysis, which included assessing conventional nutritional composition, amino acids, fatty acids, and trace elements within one month. Fig. 1 illustrates the experimental workflow, detailing the comparative analysis of breast muscle composition, covering amino acids, fatty acids, and trace elements, in both Yuzhong pigeons and European meat pigeons.

### 2.4 Conventional nutritional compositions analysis

The moisture content of the squab breast muscle was determined using the direct drying method, following the national standard GB 5009.3, 2016, Determination of Moisture in Food under the National Standard for Food Safety. The crude protein content was measured using the Kjeldahl nitrogen method, as specified in GB5009.5–2016, Determination of Protein in Food Standards of National Food Safety. The crude fat content was assessed via the Soxhlet extraction method according to GB 5009.6, 2016, Determination of Fat in Food under the National Standard for Food Safety.

### 2.5 Amino acid analysis

The amino acid content in the breast muscle of squabs was determined based on the method of Liang et al., using high-performance liquid chromatography (Liang et al., 2024). For sample hydrolysis, 0.2–0.5 g of the mixed sample was weighed and combined with 10 mL of hydrochloric acid solution (6 mol/L) in a hydrolysis tube. The tube was sealed and placed in an electric thermostat at 110 ± 1 °C for 22 h. After cooling to room temperature, the sample was filtered with water 6–7 times and then adjusted to a final volume of 50 mL in a volumetric flask. For the amino acid analysis, both the amino acid standard solution and the sample solution were injected into the amino acid analyzer at equal volumes. The amino acid concentration in the sample was calculated by comparing the peak area, using the external standard method.

### 2.6 Fatty acid analysis

The fatty acid content in the breast muscle of squabs was analyzed following the method described by Wu and Ji (2022). An appropriate amount of the mixed sample was weighed, and 10 mL of hydrochloric acid solution (8.3 mol/L) was added to a flask. The flask was placed in a 70–80 °C water bath for 40 min, with occasional shaking. After hydrolysis was complete, the sample was cooled to room temperature. To the hydrolyzed sample, 10 mL of 95 % ethanol was added and mixed thoroughly. The mixture was transferred to a separatory funnel, and the flask and stopper were rinsed with 50 mL of an ether-petroleum ether mixture. The rinses were combined in the separatory funnel, shaken for 5 min, and allowed to stand for 10 min. The ether layer was collected into a 250 mL flask. This extraction process was repeated three times, and the combined ether layers were concentrated to dryness using a rotary evaporator to obtain a fat extract. Eight mL of 2 % sodium hydroxide methanol solution was added to the fat extract, and the mixture was subjected to reflux in a water bath at 80 ± 1 °C until oil droplets were no longer visible. Subsequently, 7 mL of 15 % boron trifluoride solution was added, and the reflux process was continued for an additional 2 min at the same temperature. The condenser was rinsed with a small amount of water before stopping the heating. The flask was then removed and cooled to room temperature. Next, 10–30 mL of n-heptane was added and shaken for 2 min. A saturated sodium chloride solution was then introduced, and the mixture was left to stand for phase separation. Five mL of the upper layer was transferred to a test tube, followed by the addition of 3–5 g of anhydrous sodium sulfate, shaken for 1 min, and allowed to stand for 5 min. The upper solution was then drawn into an injection vial for analysis. The mixed fatty acid methyl ester standard solution and sample solution were injected into a gas chromatograph. Fatty acids were identified by retention time and quantified by peak area using the external standard method.

### 2.7 Trace element analysis

The total content of copper (Cu), zinc (Zn), and iron (Fe) in the breast muscle was determined using total reflection X-ray fluorescence spectrometry (S4 T-STAR, Bruker, USA), as described by Meng et al. (Meng et al., 2024). For the copper (Cu), zinc (Zn), and iron (Fe) sample digestion: a sample weighing 0.45 g was prepared, and for samples with high water content, the weight was appropriately increased to 1 g. Alternatively, 2.00 mL of liquid sample was accurately transferred into the microwave digestion inner tank. Nitric acid (6 mL) was added, the tank was covered, and the sample was left to stand for 1 h. The tank cover was tightened, and digestion was performed according to the standard operating procedures of the microwave digestion instrument. After cooling, the digestion tank was removed, and the tank cover was slowly opened to allow for exhaust. The inner cover was rinsed with a small amount of water, and the digestion tank was placed on a temperature-controlled electric hot plate, heated at 100 °C for 30 min. The solution was then diluted to 25 mL with water, mixed well for use, and a blank test was performed simultaneously. Preparation of Standard Curve: A standard series working solution was injected into the inductively coupled plasma emission spectrometer. The intensity signal response value of the analysis line for the element of interest was measured. A standard curve was prepared with the concentration of the element as the horizontal coordinate and the intensity response value of its analysis line as the vertical coordinate. Determination of Sample Solution: The blank solution and sample solution were injected into the inductively coupled plasma emission spectrometer. The signal response value of the analysis line intensity for the element of interest was determined, and the concentration of the element in the digestion solution was calculated using the standard curve.

Selenium (Se) content was measured following the method of Juszczak-Czasnojć et al. using a Shimadzu RF-5001 PC spectrofluorophotometer (Shimadzu, Duisburg, Germany) (Juszczak-Czasnojć et al., 2023). Selenium (Se) sample digestion: A 2.0 g mixed sample was weighed and placed in a conical flask. A mixture of 10 mL nitric acid and perchloric acid (5 + 1) was added for digestion until the solution became clear and white smoke was observed. After cooling, 5 mL of hydrochloric acid solution (6 mol/L) was added, and digestion was continued until the flask was filled with white smoke. The digested solution was transferred to a 10 mL volumetric flask after cooling, followed by the addition of 2.5 mL potassium ferrocyanide solution. The solution was diluted with water, shaken well, and a blank experiment was conducted simultaneously. Then, for the sample determination: A suitable working curve was prepared, with a general concentration range of 0.5–10 ng/mL. Samples were injected sequentially from low to high concentration, and fluorescence intensity was measured. A standard curve was plotted with fluorescence intensity as the ordinate and concentration as the abscissa. Under the same experimental conditions as the standard determination, the blank and test solutions were introduced and removed, respectively, and fluorescence intensity was measured. The selenium content in the sample was calculated using the standard curve.

### 2.8 Statistical analysis

All data were expressed as means ± standard deviation (S.D.) and analyzed using SPSS 27.0 (Chicago, IL, USA). The nutrient composition data of the breast muscle from the two squab breeds were statistically evaluated using a *t*-test. To further explain differences in conventional nutritional composition, including proximate composition, amino acids, fatty acids, and trace elements between Yuzhong pigeons and European meat pigeons, principal component analysis (PCA) was performed using the covariance matrix in OriginPro 2017c (Northampton, MA, USA). The same statistical method was applied to analyze the nutritional composition of breast muscle. To meet PCA requirements for linear relationships (Wu & Ji, 2022), fatty acid composition data were transformed using sine (cosine) or logarithmic conversion to ensure variance homogeneity. Factor extraction in PCA was based on eigenvalues greater than 0.5. The Pearson correlation coefficient method was employed to assess correlations between various indicators of amino acid and fatty acid compositions. Mantel test analysis was conducted using the Mantel function in R software (version 4.0.3), which calculates correlations between two matrices and provides corresponding *r* and *p* values. Differences were considered not significant at *P* > 0.05, significant at *P* < 0.05, and highly significant at *P* < 0.01.

## 3 Results

### 3.1 Comparison of conventional nutritional components of breast muscle between Yuzhong pigeons and European meat pigeons

**Fig. 2:**
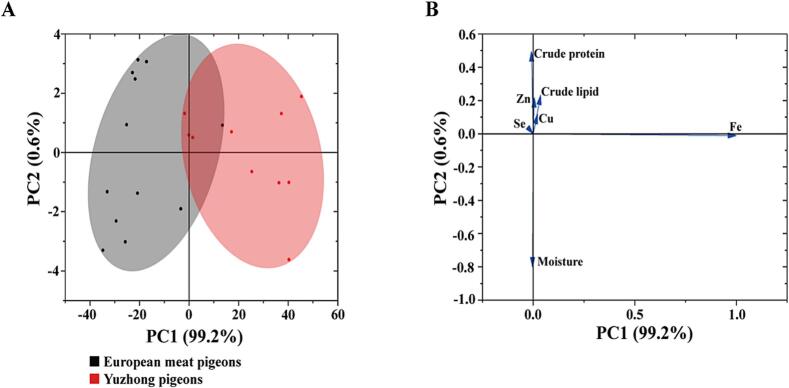
PCA score plot (A) and loading plot (B) of proximate composition and trace elements composition between Yuzhong pigeons and European meat pigeons in breast muscle. Notes: A total of 7 nutritional component variables were selected for analysis. PC1, PC2, and PC3 represent principal components 1, 2, and 3, respectively. The values in Table 5 represent loadings, which indicate the degree and direction of the relationship between each variable and its corresponding principal component. Significant loading values (>0.5) are highlighted in bold, with (+) and (−) signs representing positive and negative correlations, respectively.

The conventional nutritional components of breast muscle between Yuzhong pigeons and European meat pigeons show relatively minimal differences. Both pigeon breeds exhibit similar moisture content, with European meat pigeons at 74.17 g/100 g and Yuzhong pigeons slightly lower at 73.87 g/100 g. The protein content is also comparable, with European meat pigeons showing 21.23 g/100 g and Yuzhong pigeons slightly less at 20.82 g/100 g. The fat content is slightly higher in Yuzhong pigeons (3.82 g/100 g) compared to European meat pigeons (3.64 g/100 g). Overall, these results suggest that both breeds offer similar nutritional profile, particularly in terms of moisture, protein, and fat, with only slight variations that may not significantly impact their overall nutritional value for human consumption. However, the slight increase in fat content in Yuzhong pigeons might contribute to a richer flavor, which could be of interest to consumers. These findings underscore the potential of both breeds as valuable sources of high-quality protein with minimal fat content, making them suitable for health-conscious diets. Table 2 summarizes the comparison of common nutritional components in the breast muscle between Yuzhong pigeons and European meat pigeons. Fig. 2 illustrates the PCA score plot (A) and loading plot (B) of the selected nutritional component variables derived from the analysis of the proximate composition in the breast muscle of Yuzhong pigeons and European meat pigeons.

**Table 2:** Comparison of breast muscle proximate composition between Yuzhong pigeons and European meat pigeons (% wet matter).

Breeds	European meat pigeons	Yuzhong pigeons	*P value*
Moisture	74.17 ± 2.00	73.87 ± 1.22	0.660
Crude protein	21.23 ± 1.28	20.82 ± 1.17	0.405
Crude lipid	3.64 ± 0.79	3.82 ± 0.61	0.544

Note: The same shoulder label or no shoulder label between peer data indicates no significant difference (*P* < 0.05), the shoulder label between peer data with uppercase letters indicates a significant difference (*P* < 0.01), and the shoulder label between peer data with lowercase letters indicates a significant difference (*P* < 0.05).

### 3.2 Comparison of amino acid composition of breast muscle between Yuzhong pigeons and European meat pigeons

**Fig. 3:**
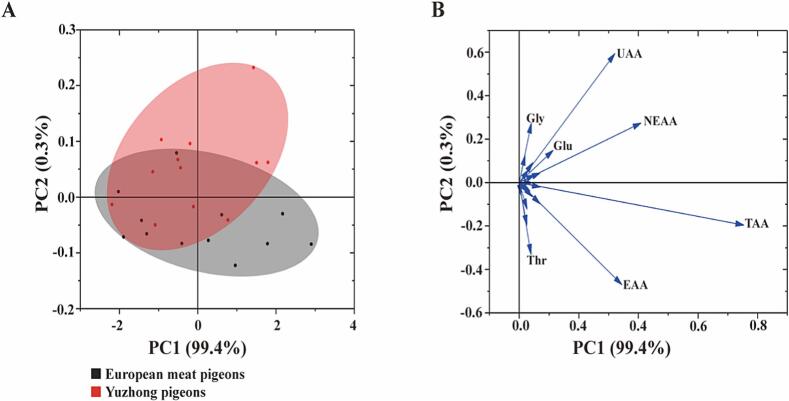
PCA score plot (A) and loading plot (B) of the amino acid composition between Yuzhong pigeons and European meat pigeons in breast muscle. Notes: A total of 21 amino acid variables were selected for analysis. PC1, PC2, and PC3 represent principal components 1, 2, and 3, respectively. The values in Table 6 represent loadings, indicating the degree and direction of the relationship between each variable and the corresponding principal component. Significant loading values (>0.5) are highlighted in bold, with (+) and (−) signs denoting positive and negative correlations, respectively.

Table 3, which compares the breast muscle amino acid composition between Yuzhong pigeons and European meat pigeons, highlights several noteworthy similarities and differences. Total amino acids (TAA) in both breeds are quite similar, with European meat pigeons at 19.68 g/100 g and Yuzhong pigeons at 19.52 g/100 g, indicating that the overall amino acid content is consistent between the two. For essential amino acids (EAA), no significant differences are observed in most of the components, such as lysine, phenylalanine, isoleucine, leucine, valine, methionine, and histidine. However, threonine is significantly lower in Yuzhong pigeons (0.98 g/100 g) compared to European meat pigeons (1.04 g/100 g) (*P* < 0.05). The total EAA and the percentage of EAA to TAA (EAA%) between the two breeds are very close, with European meat pigeons at 8.68 g/100 g (44.10 %) and Yuzhong pigeons at 8.59 g/100 g (44.02 %). For non-essential amino acids (NEAA), Yuzhong pigeons have slightly lower levels of serine and cysteine compared to European meat pigeons (*P* < 0.05). However, in other NEAAs like aspartic acid, glutamic acid, glycine, alanine, tyrosine, arginine, and proline, the two breeds show no significant differences. Interestingly, Yuzhong pigeons have a higher proportion of umami amino acids (UAA), which includes aspartic acid and glutamic acid. The UAA percentage is significantly higher in Yuzhong pigeons (44.06 %) than in European meat pigeons (43.66 %) (*P* < 0.05). Table 4 summarizes PCA analysis results based on amino acid composition in the breast muscle of Yuzhong pigeons and European meat pigeons. The comparison between the two varieties revealed significant differences in amino acid composition (*P* < 0.05). According to Table 4, the PCA of breast muscle amino acids demonstrated that the cumulative contribution rate of the first three principal components (PC1, PC2, and PC3) exceeded 95 %, indicating that these three factors effectively represent the 21 amino acids analyzed. When the loading diagrams for amino acid composition were examined (Fig. 3), it was observed that total amino acids (TAA) and umami amino acids (UAA) had a major influence on the overall amino acid composition, as indicated by principal component loading values greater than 0.5. However, no significant differences were observed in the TAA and UAA content between Yuzhong pigeons and European meat pigeons (*P* > 0.05).

**Table 3:** Comparison of breast muscle amino acid composition between Yuzhong pigeons and European meat pigeons (% wet matter).

Amino acids	European meat pigeons	Yuzhong pigeons	*P value*
Lys	1.85 ± 0.12	1.84 ± 0.09	0.754
Phe	0.90 ± 0.06	0.90 ± 0.04	1.000
Thr	1.04 ± 0.07 ^a^	0.98 ± 0.05 ^b^	0.033
Ile	1.02 ± 0.07	1.01 ± 0.05	0.723
Leu	1.72 ± 0.12	1.72 ± 0.08	0.859
Val	1.07 ± 0.07	1.07 ± 0.05	0.975
Met	0.56 ± 0.04	0.55 ± 0.03	0.692
His	0.53 ± 0.04	0.54 ± 0.04	0.645
EAA	8.68 ± 0.58	8.59 ± 0.41	0.681
EAA %	44.10 % ± 0.26 %	44.02 % ± 0.18 %	0.399
Asp*	1.82 ± 0.11	1.81 ± 0.09	0.782
Ser	0.67 ± 0.04 ^A^	0.62 ± 0.03 ^B^	0.004
Glu*	3.09 ± 0.18	3.09 ± 0.15	0.904
Gly*	1.07 ± 0.07	1.10 ± 0.06	0.269
Ala*	1.24 ± 0.07	1.25 ± 0.06	0.718
Cys	0.22 ± 0.01 ^A^	0.21 ± 0.01 ^B^	0.006
Tyr	0.66 ± 0.04	0.64 ± 0.03	0.156
Arg*	1.37 ± 0.08	1.36 ± 0.06	0.830
Pro	0.86 ± 0.06	0.86 ± 0.04	0.903
UAA	8.59 ± 0.52	8.60 ± 0.42	0.945
UAA%	43.66 % ± 0.18 % ^B^	44.06 % ± 0.24 % ^A^	0.000
NEAA	11.00 ± 0.66	10.93 ± 0.52	0.757
NEAA %	55.92 ± 0.26	55.98 ± 0.17	0.504
TAA	19.68 ± 1.24	19.52 ± 0.92	0.733

Note: 1* indicates umami amino acid. 2 The same shoulder label or no shoulder label among peer data indicates no significant difference (*P* < 0.05); uppercase letter of shoulder label among peer data indicates significant difference (*P* < 0.01); lowercase letter of shoulder label among peer data indicates significant difference (*P* < 0.05). 3 NEAA: Non-essential amino acids, EAA: Essential amino acids, TAA: Total amino acids, UAA: umami amino acids.

**Table 4:** Eigen analysis of the covariance matrix loadings for significant principal components for amino acids between Yuzhong pigeons and European meat pigeons.

Contents	Factor 1	Factor 2	Factor 3
Phe	0.036	−0.040	0.133
Met	0.024	−0.041	0.054
Lys	0.070	−0.021	0.023
Leu	0.070	−0.098	0.180
Thr	0.039	−0.331	−0.367
Val	0.043	−0.001	0.183
Ile	0.043	−0.064	0.073
His	0.020	0.127	0.199
EAA	0.344	−0.470	0.478
Ala	0.045	0.092	0.122
Gly	0.041	0.274	0.081
Glu	0.114	0.148	−0.016
Arg	0.051	0.035	−0.002
Asp	0.070	0.044	0.029
Tyr	0.026	−0.123	−0.128
Pro	0.031	0.058	−0.103
Ser	0.025	−0.197	−0.317
Cys	0.005	−0.058	−0.187
UAA	0.321	0.594	0.213
NEAA	0.409	0.274	−0.522
TAA	0.753	−0.196	−0.045
Eigenvalue	2.02	0.01	0.00
Percentage of Variance/%	99.42	0.34	0.10
Cumulative/%	99.42	99.75	99.85

Both pigeon breeds have a similar overall amino acid composition, Yuzhong pigeons exhibit a higher proportion of umami amino acids, which may contribute to their unique flavor profile. On the other hand, European meat pigeons have slightly higher levels of threonine, which is essential for protein synthesis and overall muscle development. The findings suggest that both breeds offer a nutritionally valuable amino acid profile, but with subtle differences that could influence flavor and dietary benefits. These variations provide important insights for breeders and consumers looking for specific nutritional or culinary traits in pigeon meat.

### 3.3 Comparison of fatty acid composition of breast muscle between Yuzhong pigeons and European meat pigeons

Table 5 summarizes the comparison of fatty acids composition in the breast muscle between Yuzhong pigeons and European meat pigeons. The breast muscle of Yuzhong pigeons and European meat pigeons demonstrates distinct differences in fatty acid profiles, offering valuable insights into their nutritional qualities. For the saturated fatty acids (SFAs), both breeds exhibit similar levels of saturated fatty acids (SFAs), with European meat pigeons at 31.95 % and Yuzhong pigeons slightly higher at 32.43 %. The most prevalent SFAs, such as palmitic acid (C16:0) and stearic acid (C18:0), show no substantial differences between the breeds. However, eicosanoic acid (C20:0) is significantly lower in Yuzhong pigeons (*P* < 0.05), which may indicate a slight variation in fat metabolism between the breeds. In the Monounsaturated Fatty Acids (MUFAs) analysis, the results indicate that Yuzhong pigeons have significantly higher monounsaturated fatty acids (MUFAs) at 39.60 %, compared to 34.87 % in European meat pigeons (P < 0.05). This difference is largely driven by the higher concentration of oleic acid (C18:1n9) in Yuzhong pigeons, which is known for its health benefits, including improving heart health and reducing inflammation. This suggests that Yuzhong pigeons may offer greater nutritional value in terms of healthy fats, making them potentially more appealing for health-conscious consumers.

**Table 5:** Comparison of breast muscle fatty acids composition between Yuzhong pigeons and European meat pigeons (% wet matter).

Fatty acids	European meat pigeons	Yuzhong pigeons	*P value*
C14:0	0.32 ± 0.06	0.32 ± 0.02	0.890
C16:0	20.42 ± 0.66	20.54 ± 1.09	0.798
C18:0	10.67 ± 1.39	11.11 ± 1.13	0.429
C20:0	0.11 ± 0.02 ^A^	0.08 ± 0.01 ^B^	0.001
C22:0	0.43 ± 0.09	0.38 ± 0.06	0.177
SFA	31.95 ± 1.53	32.43 ± 1.44	0.482
C14:1n-5	0.10 ± 0.06	0.09 ± 0.03	0.586
C16:1n-5	6.45 ± 1.93	6.76 ± 1.46	0.580
C18:1n-9	27.78 ± 2.62 ^B^	32.29 ± 3.23 ^A^	0.004
C20:1n-9	0.22 ± 0.06	0.20 ± 0.03	0.442
C24:1n-9	0.31 ± 0.09	0.26 ± 0.06	0.145
MUFA	34.87 ± 4.09 ^b^	39.60 ± 4.31 ^a^	0.023
C18:2n-6	24.88 ± 2.13 ^A^	21.18 ± 2.22 ^B^	0.002
C20:2n-6	0.23 ± 0.06 ^a^	0.16 ± 0.03 ^b^	0.011
C20:4n-6	6.49 ± 1.05 ^a^	5.38 ± 1.10 ^b^	0.031
n-6PUFA	31.59 ± 2.66 ^A^	26.72 ± 3.31 ^B^	0.003
C18:3n-3	0.51 ± 0.14 ^A^	0.35 ± 0.05 ^B^	0.001
C20:5n-3	0.22 ± 0.04 ^A^	0.14 ± 0.03 ^B^	<0.001
C22:6n-3	0.28 ± 0.07 ^a^	0.22 ± 0.06 ^b^	0.043
n-3PUFA	1.01 ± 0.15 ^A^	0.72 ± 0.12 ^B^	<0.001
PUFA	32.60 ± 2.73 ^A^	27.44 ± 3.43 ^B^	0.002
n-3/n-6PUFA	0.032 ± 0.004	0.027 ± 0.002	0.003

Note: The same shoulder label or no shoulder label between peer data indicates no significant difference (*P* < 0.05), the shoulder label between peer data with uppercase letters indicates a significant difference (*P* < 0.01), and the shoulder label between peer data with lowercase letters indicates a significant difference (*P* < 0.05). SFA: saturated fatty acid, MUFA: monounsaturated fatty acid, HUFA: highly unsaturated fatty acid, PUFA: poly-unsaturated fatty acid.

**Fig. 4:**
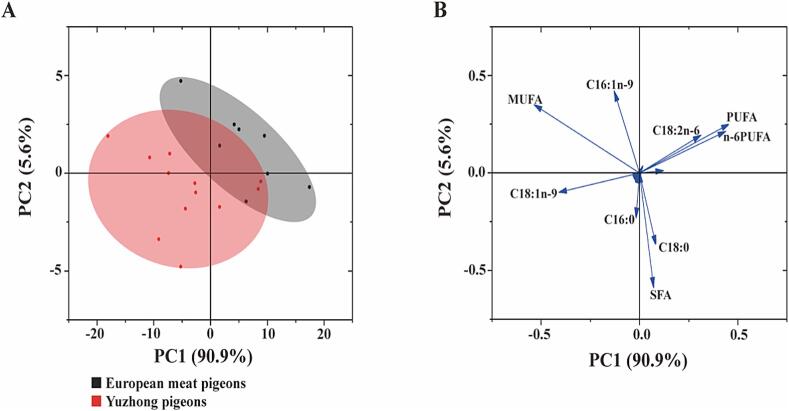
PCA score plot (A) and loading plot (B) of the fatty acid composition between Yuzhong pigeons and European meat pigeons in breast muscle. Notes: A total of 22 fatty acid variables were selected for analysis. PC1, PC2, and PC3 represent principal components 1, 2, and 3, respectively. The values in Table 7 denote the loadings, which indicate the degree and direction of the relationship between each variable and the corresponding principal component. Significant loading values (>0.5) are highlighted in bold. Positive and negative correlations are represented by (+) and (−) signs, respectively.

In the Polyunsaturated Fatty Acids (PUFAs) analysis, European meat pigeons show significantly higher polyunsaturated fatty acids (PUFAs) overall (32.60 %) compared to Yuzhong pigeons (27.44 %) (P < 0.05). This includes higher levels of key n6-PUFAs such as linoleic acid (C18:2n6) and arachidonic acid (C20:4n6). In addition, European meat pigeons have higher concentrations of n3-PUFAs, such as linolenic acid (C18:3n3), EPA (C20:5n3), and DHA (C22:6n3), all of which are essential for cardiovascular health, brain function, and anti-inflammatory effects. Both pigeon breeds exhibit a similar and low n3/n6-PUFA ratio (0.03), indicating that while they offer beneficial fatty acids, their balance between omega-3 and omega-6 is relatively low, which may have implications for inflammation management and overall dietary balance. Both pigeon breeds offer distinct nutritional benefits in terms of fatty acid composition. Yuzhong pigeons, with higher MUFAs, may provide superior cardiovascular health benefits, while European meat pigeons, richer in PUFAs, offer essential fatty acids that support broader health functions. These results could guide dietary choices and breeding programs aimed at optimizing the nutritional quality of pigeon meat. Table 6 summarize PCA analysis results based on fatty acids composition in breast muscle between Yuzhong pigeons and European meat pigeons. Comparison of two varieties reveals that significant differences were observed in fatty acids composition (*P* < 0.05). According to Table 6, PCA of breast muscle demonstrated that cumulative contribution rate of the first three common factors is over 95 %, indicating that the above three main factors (PC1, PC2, and PC3) can be used to represent the 22 kinds of fatty acids composition. When the loading diagrams of fatty acids composition in breast muscle were combined in Fig. 4, we found that the MUFA and SFA composition in breast muscle had a major impact on the fatty acids composition (the characteristic value of principal component loading value >0.5) and that the SFA composition in breast muscle had no significantly difference between Yuzhong pigeons and European meat pigeons (*P* > 0.05), the MUFA content in breast muscle of European meat pigeons and Yuzhong pigeons had a significantly increased than Yuzhong pigeons (*P* < 0.05).

**Table 6:** Eigen analysis of the covariance matrix loadings for significant principal components for fatty acids between Yuzhong pigeons and European meat pigeons.

Contents	Factor 1	Factor 2	Factor 3
C14:0	−0.001	0.006	−0.006
C16:0	−0.018	−0.233	−0.422
C18:0	0.084	−0.374	0.244
C20:0	0.001	0.005	−0.003
C22:0	0.005	0.007	−0.015
SFA	0.072	−0.589	−0.203
C14:1n-5	−0.001	0.014	−0.014
C16:1n-5	−0.129	0.421	−0.492
C18:1n-9	−0.411	−0.099	0.594
C20:1n-9	−0.001	0.009	−0.006
C24:1n-9	0.005	0.005	−0.017
MUFA	−0.536	0.350	0.065
C18:2n-6	0.313	0.194	0.262
C20:2n-6	0.004	0.008	−0.014
C20:4n-6	0.122	0.012	−0.096
n-6PUFA	0.439	0.213	0.152
C18:3n-3	0.007	0.023	0.008
C20:5n-3	0.004	0.011	−0.008
C22:6n-3	0.007	0.003	−0.010
n-3PUFA	0.018	0.038	−0.011
PUFA	0.457	0.251	0.142
n-3/n-6PUFA	0.000	0.001	−0.001
Eigenvalue	76.42	4.72	2.00
Percentage of Variance/%	90.86	5.61	2.37
Cumulative/%	90.86	96.47	98.84

### 3.4 Comparison of trace elements in breast muscle between Yuzhong pigeons and European meat pigeons

The comparison of breast muscle microelement components between Yuzhong pigeons and European meat pigeons shows significant differences in key microelements that are important for human health. Selenium levels are higher in Yuzhong pigeons (0.24 mg/kg) compared to European meat pigeons (0.18 mg/kg) (*P* < 0.05) (Table 7). Selenium is an essential trace element known for its role in antioxidant activity and immune function. The copper content in Yuzhong pigeons (4.48 mg/kg) is significantly higher than in European meat pigeons (3.38 mg/kg) (*P* < 0.05). Zinc, crucial for immune function, protein synthesis, and wound healing, is found in higher concentrations in Yuzhong pigeons (12.83 mg/kg) than in European meat pigeons (11.00 mg/kg) (*P* < 0.05). The most striking difference is observed in iron levels, with Yuzhong pigeons containing almost twice the amount (101.85 mg/kg) compared to European meat pigeons (57.68 mg/kg) (*P* < 0.05). The significantly higher levels of selenium, copper, zinc, and iron in Yuzhong pigeons compared to European meat pigeons suggest that Yuzhong pigeons provide a more nutrient-dense option in terms of trace minerals. Yuzhong pigeons appear to offer enhanced nutritional value in terms of essential trace minerals compared to European meat pigeons. These findings are important for dietary recommendations and could inform breeding programs aimed at improving the nutritional quality of pigeon meat for human consumption. Table 8 summarizes PCA analysis results based on proximate composition and trace element composition in the breast muscle of Yuzhong pigeons and European meat pigeons, showing significant differences between the two varieties (*P* < 0.05). According to Table 5, the PCA of breast muscle demonstrated that the cumulative contribution rate of the first three principal components (PC1, PC2, and PC3) exceeded 95 %, indicating that these three factors effectively represent the seven proximate and trace element components analyzed. From the loading diagrams (Fig. 2), it was observed that the iron (Fe) content in breast muscle had the most significant influence on the overall proximate and trace element composition, as indicated by principal component loading values greater than 0.5. Additionally, the Fe content in the breast muscle of Yuzhong pigeons was significantly higher than that of European meat pigeons (*P* < 0.05).

**Table 7:** Comparison of breast muscle common mineral element composition between Yuzhong pigeons and European meat pigeons (% wet matter).

Item(mg/kg)	European meat pigeons	Yuzhong Pigeons	*P value*
Se	0.18 ± 0.02 ^B^	0.24 ± 0.04 ^A^	0.000
Cu	3.38 ± 0.63 ^B^	4.48 ± 0.38 ^A^	0.000
Zn	11.00 ± 1.37 ^B^	12.83 ± 0.75 ^A^	0.001
Fe	57.68 ± 13.31 ^B^	101.85 ± 18.33 ^A^	0.000

**Table 8:** Eigen analysis of the covariance matrix loadings for significant principal components for proximate and mineral elements between Yuzhong pigeons and European meat pigeons.

Contents	Factor 1	Factor 2	Factor 3
Moisture	−0.004	−0.801	0.023
Crude protein	−0.009	0.494	−0.497
Crude lipid	0.005	0.217	0.209
Fe contents	0.999	−0.011	−0.041
Cu contents	0.021	0.118	0.323
Zn contents	0.037	0.232	0.777
Se contents	0.001	−0.002	−0.011
Eigenvalue	749.55	4.31	1.17
Percentage of Variance/%	99.19	0.57	0.15
Cumulative/%	99.19	99.76	99.92

### 3.5 Correlation analysis of nutritional composition in breast muscle between Yuzhong pigeons and European meat pigeons

**Fig. 5:**
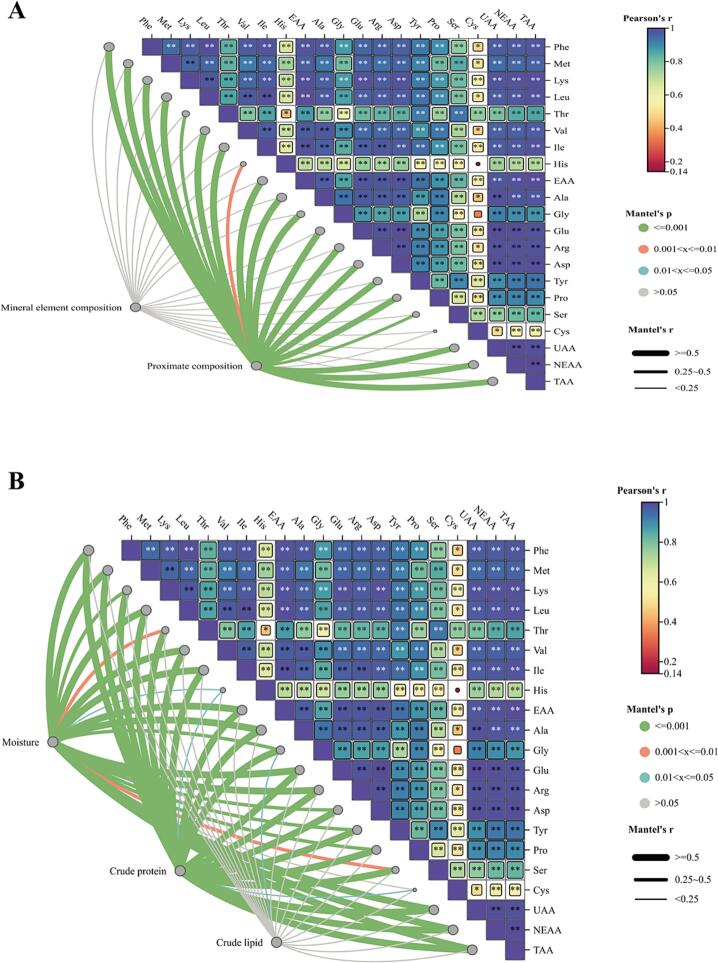
Correlation analysis of proximate composition, trace elements composition, and amino acids composition in breast muscle between Yuzhong pigeons and European meat pigeons. Heat map of multidimensional correlation between proximate composition, trace elements composition and amino acids composition of the breast muscle (A). Heat map of multidimensional correlation between moisture, crude protein, crude lipid and amino acids composition of the breast muscle (B). Note: The existence of “*” and “**” indicates a significant difference between the two comparisons. The “ns” stands for no significant difference, *P < 0.05, ** *P* < 0.01, respectively (*n* = 12 replicate pigeons). Proximate composition and trace element composition were compared pairwise with amino acid composition, where the color gradient and block size reflect Pearson's correlation coefficients (based on Euclidean distance). Partial Mantel's tests were used to analyze the relationship between proximate composition, trace element composition, and amino acid composition. The edge width corresponds to the Mantel's r statistic for distance correlations, and the edge color indicates statistical significance based on 9999 permutations.

**Fig. 6:**
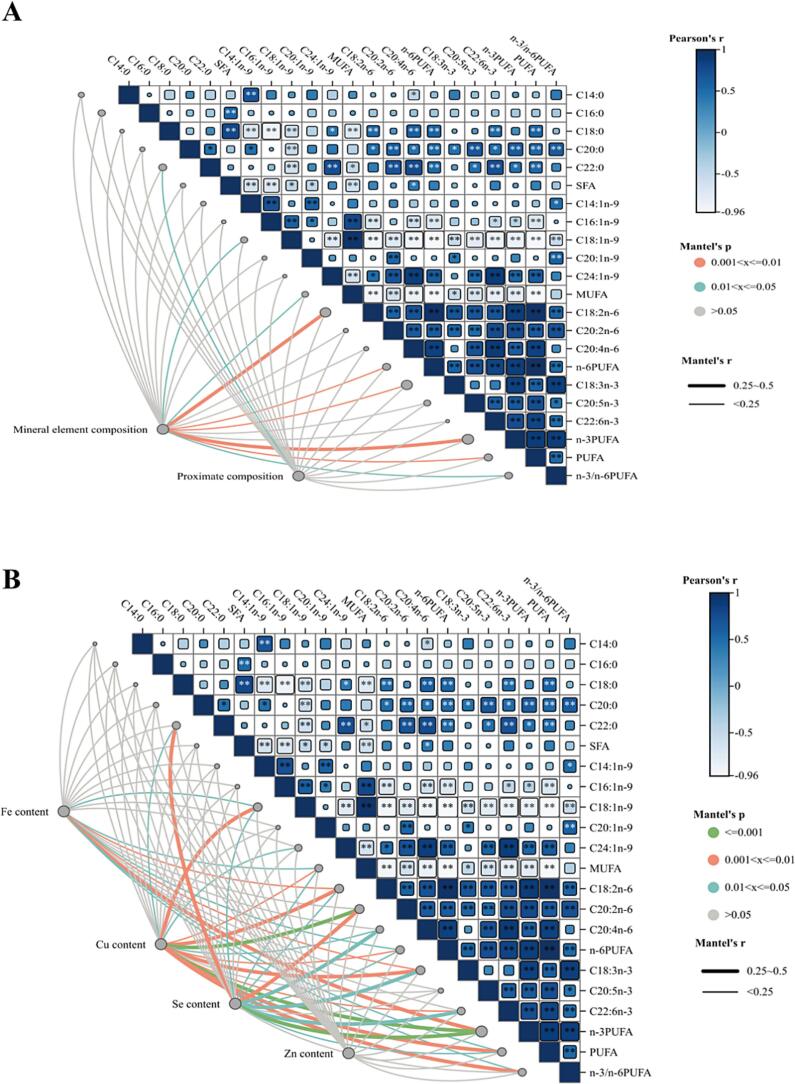
Correlation analysis of proximate composition, trace elements composition and amino acids composition in breast muscle between Yuzhong pigeons and European meat pigeons. Heat map of multidimensional correlation between proximate composition, trace elements composition and fatty acids composition of the breast muscle (A). Heat map of multidimensional correlation between Fe, Cu, Se and Zn content of the breast muscle (B). Note: The existence of “*” and “**” indicates a significant difference between the two comparisons. The “ns” stands for no significant difference, *P < 0.05, ** *P* < 0.01, respectively (n = 12 replicate pigeons). Proximate and trace element compositions were compared pairwise with fatty acid composition, with the color gradient and block size reflecting Pearson's correlation coefficients (based on Euclidean distance). Partial Mantel's tests were employed to relate proximate composition and trace element composition to fatty acid composition. The edge width corresponds to Mantel's r statistic for distance correlations, and edge color indicates statistical significance based on 9999 permutations.

Through correlation analysis, we observed that the amino acid composition in breast muscle is strongly correlated with the proximate composition, showing highly significant relationships. Analysis of the correlation between each conventional component and amino acid composition (Fig. 5A) revealed that moisture and crude protein content were extremely significantly correlated with amino acid composition (*P* < 0.05), whereas crude lipid showed no such significant correlation (*P* > 0.05) (Fig. 5B). Conversely, further correlation analysis demonstrated a strong and highly significant correlation between fatty acid composition and trace element composition in breast muscle (*P* < 0.05). However, no significant correlation was found between fatty acid composition and proximate composition. By analyzing the correlation between trace elements and proximate composition (Fig. 6A), selenium (Se) and copper (Cu) showed an extremely significant correlation with fatty acid composition (*P* < 0.05), while zinc (Zn) did not exhibit a strong correlation (*P* > 0.05). The correlation of iron (Fe) with fatty acid composition in breast muscle was intermediate, lying between that of Se, Cu, and Zn (Fig. 6B).

## 4 Discussion

### 4.1 Conventional nutritional components in breast muscle of meat pigeons

Pigeon meat is recognized for its high nutritional value, characterized by low fat content and high digestibility, making it a desirable source of protein for human consumption (Chang, Tang, et al., 2023; Chang, Zhang, et al., 2023). Due to its favorable chemical composition, pigeon meat is often recommended for health-conscious individuals and those seeking lean protein options. Understanding the nutritional makeup of pigeon muscle, particularly in terms of conventional components such as water, crude protein, and crude fat, is essential for evaluating its value as a food source (Winiarska-Mieczan et al., 2016).

From a protein perspective, meat pigeons compare favorably with other traditional meat sources. For example, protein content in pork, beef, and mutton averages around 20.3 %, 20.1 %, and 20.5 %, respectively (Eom et al., 2024; Siddiqui et al., 2024; Sun & Jiang, 2022). Poultry meat, in general, has a protein content range of 17–25 % (Tay-Zar et al., 2023). Several studies have specifically examined the protein content of pigeon breeds. Pomianowski et al. found that three breeds of meat pigeons exhibited protein content between 21.73 % and 23.61 % (Pomianowski et al., 2009). Chang Lingling et al. reported that European meat pigeon squabs had a protein content of 20.5 % (Chang et al., 2017), while Tang Qingping et al. identified a protein content of 21.79 % in Wugang pigeons (Tang et al., 2019). In the current study, the protein content of European meat pigeons was 21.24 %, slightly higher than that of Yuzhong pigeons, which had a protein content of 20.82 %. This aligns with previous findings and reinforces the conclusion that both breeds offer substantial protein levels comparable to other high-quality meat sources.

Fat content is another critical factor in assessing the nutritional value of meat pigeons. Pigeon meat is known for being relatively lean, which enhances its appeal to health-conscious consumers. Pomianowski et al. found fat content ranging from 4.32 % to 7.07 % in different meat pigeon breeds (Pomianowski et al., 2009). In contrast, Chang Lingling et al. reported a much lower fat content of 2.7 % in European meat pigeons (Chang et al., 2017), and Tang Qingping et al. found a similar fat content of 2.57 % in Wugang pigeons (Tang et al., 2019). In the present study, the fat content of Yuzhong pigeons was measured at 3.82 %, while European meat pigeons had a slightly lower fat content of 3.64 %. Although these values are higher than those reported in some previous studies, they confirm that both breeds remain low-fat options, which is an important consideration for those seeking healthier meat alternatives.

Water content plays a significant role in the overall quality and tenderness of meat, especially in young pigeons, or squabs, that are in the early stages of growth. Higher water content is typical at this stage and can influence both texture and perceived quality. Additionally, research has shown that increased water content in muscle is generally associated with higher levels of flavorful amino acids and polyunsaturated fatty acids (Li et al., 2024). This may occur because water helps maintain the structure and stability of flavorful amino acids (Chu et al., 2024). The water content in the breast muscle of European meat pigeons was 74.17 %, while Yuzhong pigeons had a water content of 73.87 %. These findings are consistent with previous studies, such as Chang Lingling et al. (73.20 %) (Chang et al., 2017), Tang Qingping et al. (73.51 %) (Tang et al., 2019), and Bu Zhu et al. (72.5–72.99 %) (Bu et al., 2018). These water content values are slightly higher than those reported by Pomianowski et al., who found water content between 66.52 % and 70.59 % in other meat pigeon breeds (Pomianowski et al., 2009). The relatively high-water content observed in both Yuzhong and European meat pigeons indicates that these birds produce tender, moist meat, which is often preferred in culinary applications.

This study revealed no significant differences in the conventional nutritional components-protein, fat, and water-between Yuzhong pigeons and European meat pigeons. Both breeds demonstrated similar nutritional profiles, suggesting that Yuzhong pigeons offer a comparable level of nutritional value to the widely recognized European meat pigeon breed. This finding is particularly important for local pigeon breeders and consumers, as it highlights the potential of Yuzhong pigeons as a viable alternative to imported breeds. The comparable nutritional content of these two pigeon breeds underscores the broader value of pigeon meat as a nutritious, lean, and protein-rich option in the human diet. Such information can guide both breeding strategies and dietary recommendations, ensuring that pigeon meat continues to be promoted as a healthy, sustainable protein source.

### 4.2 Amino acid content in breast muscle of meat pigeon

Amino acids, as the fundamental units of proteins, play a crucial role in determining the nutritional quality of meat. The value of proteins is largely influenced by the amino acid composition and the relative proportions of essential and non-essential amino acids within them (van Harn et al., 2019). The assessment of meat quality, particularly in terms of its protein content, hinges on the analysis of amino acids, with essential amino acids serving as the primary indicators of a protein's nutritional level (Rikimaru & Takahashi, 2010). Essential amino acids are indispensable for maintaining various physiological processes, including metabolic regulation, tissue growth, and immune function. The concentration of these amino acids, as well as the ratio of essential to non-essential amino acids, directly affects the nutritional value of meat proteins (Xu et al., 2019).

In addition to the health-related aspects of amino acids, flavor also plays a key role in consumer preference for meat products. Certain amino acids, referred to as umami amino acids or flavor amino acids, contribute to the sensory properties of meat, enhancing its taste profile. While most amino acids lend sweet or bitter notes, a select few-such as aspartate, glutamic acid, glycine, arginine, and alanine-contribute to umami or savory flavors, which can significantly impact the palatability of meat (Chang, Tang, et al., 2023; Chang, Zhang, et al., 2023). In the current study, no significant differences were observed in the total amino acid content or the proportion of essential amino acids between Yuzhong pigeons and European meat pigeons, suggesting that both breeds offer comparable protein quality. However, a notable difference was found in the proportion of umami amino acids, with Yuzhong pigeons exhibiting a higher concentration of these flavor-enhancing amino acids. This finding implies that Yuzhong pigeons may offer a more desirable flavor profile, which could be a key factor in consumer preference for this breed.

Among the essential amino acids, threonine content was slightly lower in Yuzhong pigeons compared to European meat pigeons. Threonine is an essential amino acid that plays multiple critical roles in the body, including the synthesis of proteins, maintaining nitrogen balance, enhancing immune function, promoting growth, and facilitating wound healing and tissue repair. In this study, the threonine content in the breast muscle of European meat pigeons was measured at 1.04 g/100 g, while Yuzhong pigeons contained 0.98 g/100 g. These values are in line with previous findings by Yao Junfeng et al., who reported a threonine content of 1.03 g/100 g in pigeon meat (Yao et al., 2022). Interestingly, the threonine content in pigeon meat is approximately double that of chicken meat, which contains around 0.506 g/100 g. This higher threonine content may be one reason why studies have shown that dietary consumption of pigeon meat can promote wound healing in animal models, specifically rats (Chen et al., 2019).

The study further revealed differences in non-essential amino acids between the two pigeon breeds, particularly in the proportions of serine and cystine, as well as in the concentration of umami amino acids. These findings are consistent with previous research by Li Fuhuang, who examined the primary nutrients in the breast muscle of various squab breeds and found similar patterns in amino acid composition (Li et al., 2019). However, these results differ from those of Chang Lingling et al., who found no significant differences (*P* > 0.05) in the amino acid content, the ratio of essential amino acids, or the ratio of umami amino acids among different pigeon breeds (Chang, Tang, et al., 2023; Chang, Zhang, et al., 2023). This discrepancy may be attributed to the genetic differences among the breeds studied and the varying degrees of selective breeding they have undergone. The genetic background and breeding practices could influence the amino acid composition and other nutritional traits, leading to variability in the results observed across different studies.

This finding highlights the similarities and subtle differences in the amino acid composition of Yuzhong pigeons and European meat pigeons. While both breeds provide high-quality protein with comparable essential amino acid content, Yuzhong pigeons stand out for their higher proportion of umami amino acids, which may enhance their flavor and consumer appeal. The slightly lower threonine content in Yuzhong pigeons, though marginal, does not diminish their overall nutritional value, especially given the high levels of this essential amino acid relative to other poultry meats. These findings contribute valuable insights into the nutritional profile of pigeon breeds and offer guidance for breeding programs aimed at optimizing both the nutritional and sensory qualities of pigeon meat.

### 4.3 Fatty acid content in breast muscle of meat pigeon

Fatty acids are critical biomolecules that serve a range of essential physiological functions, from maintaining the structural integrity of cell membranes to facilitating chemical signaling pathways within the body. They play pivotal roles in promoting the development of the central nervous system, generating energy, transporting oxygen, and regulating inflammatory responses. These functions not only influence the physical properties of fats but also affect the flavor and palatability of meat (Larsson et al., 2004). However, fats rich in saturated fatty acids (SFA) are widely considered harmful when consumed in excess. High SFA intake is linked to elevated cholesterol levels and an increased risk of cardiovascular diseases, obesity, and certain cancers (Calder, 2006).

Among fatty acids, polyunsaturated fatty acids (PUFAs) such as DHA (docosahexaenoic acid), arachidonic acid, linolenic acid, and linoleic acid are essential because they cannot be synthesized by the human body and must be obtained through the diet. These fatty acids are crucial for several biological processes. n-3 polyunsaturated fatty acids are particularly beneficial for cardiovascular health and immune system function, while n-6 polyunsaturated fatty acids support cell membrane integrity and normal physiological activities when consumed in appropriate amounts. The balance between these fatty acids is key to maintaining health. Previous studies have analyzed the fatty acid content of various meat sources. For example, Huang et al. reported that the proportion of saturated fatty acids in three Chinese regional pig breeds ranged between 38.4 % and 40.2 %, highlighting the relatively high SFA content in these meats (Xu et al., 2019). Aidyn et al. observed even higher SFA levels in the muscle of Kazakhstan male turkeys, with a range of 50.67 % to 52.64 %, which is considerably high compared to other poultry (Okuskhanova et al., 2019). Similarly, Vakentini et al. studied broilers fed with glycerin monolaurate additives and found that their total SFA content ranged from 31.02 % to 34.70 % (Valentini et al., 2020).

In contrast, the current study found that the SFA content in the breast muscle of Yuzhong pigeons (31.95 %) and European meat pigeons (32.43 %) was considerably lower than that of pork and turkey but similar to broilers. This lower SFA content in both pigeon breeds suggests that pigeon meat may offer a healthier alternative to meats with higher SFA levels, particularly for individuals seeking to reduce saturated fat intake. The comparable SFA content between the two pigeon breeds also implies that both are viable options for producing lean, health-conscious poultry meat. Regarding polyunsaturated fatty acids (PUFAs), which are known for their positive health effects, Yuzhong pigeons had a higher PUFA content than European meat pigeons. PUFAs, especially n-3 and n-6 fatty acids, are essential for maintaining cardiovascular health, reducing inflammation, and supporting immune function. This distinction indicates that Yuzhong pigeons may offer superior nutritional benefits in terms of essential fatty acids. The higher PUFA content in Yuzhong pigeons enhances their appeal as a health-conscious dietary choice, particularly for consumers focused on improving heart health and reducing inflammation through diet. Dietary recommendations from leading health organizations, including the World Health Organization (WHO), Food and Agriculture Organization (FAO), and the American Heart Association, emphasize the importance of regulating fat intake. These organizations suggest that total dietary fat should account for no more than 30 % of total caloric intake. Furthermore, they advocate for a balanced ratio of saturated fatty acids (SFA), monounsaturated fatty acids (MUFA), and polyunsaturated fatty acids (PUFA) in the diet, ideally at a 1:1:1 ratio, to support optimal health outcomes (Conti et al., 2024).

The current study's findings align well with these recommendations, particularly with regard to the SFA/MUFA/PUFA ratios observed in European meat pigeons, which closely match the ideal dietary ratio. This balanced fatty acid profile makes European meat pigeons a nutritionally sound choice for consumers seeking a healthier balance of fats in their diet. In contrast, while Yuzhong pigeons had a higher PUFA content, their SFA/MUFA/PUFA ratio was slightly less ideal, though still within acceptable nutritional limits. These findings suggest that while both breeds offer health benefits, European meat pigeons may provide a more balanced fatty acid composition. It is important to note that the SFA/MUFA/PUFA ratio observed in European meat pigeons in this study differs from previous findings by Yao et al. (Yao et al., 2022). This variation could be attributed to several factors, including differences in genetic strain, age of the pigeons, diet, and feeding management practices. These factors can significantly influence the fatty acid composition in poultry meat, and further research may be needed to explore how these variables affect the nutritional quality of pigeon meat across different breeds and environments.

The fatty acid composition of Yuzhong pigeons and European meat pigeons provides valuable insights into the nutritional value of pigeon meat. Both breeds contain relatively low levels of saturated fatty acids, making them a healthier alternative to higher-SFA meats like pork and turkey. Moreover, Yuzhong pigeons stand out for their higher polyunsaturated fatty acid content, which may offer additional health benefits, particularly for cardiovascular health. European meat pigeons, on the other hand, present a more balanced SFA/MUFA/PUFA ratio, aligning closely with dietary recommendations for fat intake. These findings suggest that pigeon meat, regardless of breed, is a nutritionally valuable option for consumers seeking a lean, health-conscious source of protein and essential fatty acids. Such insights have important implications for breeding strategies and dietary recommendations, highlighting the potential of pigeon meat to contribute to a healthier, more balanced diet.

### 4.4 Trace elements content in breast muscle of meat pigeon

Trace elements are critical for maintaining various biological processes in living organisms, serving as integral components of bioactive molecules such as enzymes, hormones, and vitamins. They are involved in numerous metabolic pathways and energy-transfer processes, contributing to the overall homeostasis and metabolic health of the body (Apple et al., 2000). Trace elements like copper (Cu), iron (Fe), zinc (Zn), and selenium (Se) are essential for physiological functions such as oxygen transport, immune response, and antioxidant defense. Given their importance, the variation in trace element concentrations between different pigeon breeds warrants detailed exploration, as these differences can directly affect the nutritional value of pigeon meat.

In this study, Yuzhong pigeons exhibited significantly higher concentrations of Cu, Fe, Zn, and Se in their breast muscle compared to European meat pigeons. These elevated levels of trace elements suggest that Yuzhong pigeons may provide greater nutritional benefits, especially in terms of essential minerals that are vital for human health. The selenium content in the breast muscle of Yuzhong pigeons was measured at 0.24 mg/kg, which closely aligns with the 0.27 mg/kg reported by Tang Qingping et al. in earlier studies (Tang et al., 2019). Selenium plays an important role in antioxidant defense and immune function, and its presence in meat is crucial for supporting these physiological processes. By contrast, European meat pigeons in this study contained 0.18 mg/kg of selenium, which is notably lower than the 0.5 mg/kg found in 3-month-old European meat pigeons in a study (Yao et al., 2022). The observed variation in selenium content could be attributed to age-related factors, as previous studies have indicated that selenium levels in muscle tissue tend to increase with age. The higher selenium content in Yuzhong pigeons may also be linked to genetic factors, as differences in gene expression related to selenium absorption and deposition could explain the higher levels observed in this breed. This genetic influence on trace element metabolism highlights the importance of understanding breed-specific characteristics when evaluating the nutritional profile of pigeon meat.

In addition to selenium, the concentrations of copper and iron in Yuzhong pigeons were also significantly higher than those in European meat pigeons. Copper content in the breast muscle of Yuzhong pigeons was 4.48 mg/kg, while iron content was measured at 100.95 mg/kg. These values surpass those reported by Tang Qingping et al., who found copper and iron levels of 2.53 mg/kg and 42.67 mg/kg, respectively, in pigeon breast muscle (Tang et al., 2019). Copper is essential for various biochemical reactions, including iron metabolism and the maintenance of connective tissues, while iron is a key component of hemoglobin, necessary for oxygen transport and energy production. The increased levels of these elements in Yuzhong pigeons could enhance their value as a dietary source of these essential minerals.

From a nutritional standpoint, the higher levels of selenium, copper, and iron in Yuzhong pigeons suggest that they offer superior nutritional benefits compared to European meat pigeons, particularly for consumers seeking to increase their intake of essential trace elements. These trace elements are vital for maintaining overall health, supporting immune function, and facilitating critical metabolic processes. Selenium plays a key role in protecting the body from oxidative stress and enhancing immune response. A previous study revealed that the addition of selenium can increase the content of non-volatile n-3 polyunsaturated fatty acids and enhance meat cohesiveness (Hui et al., 2025). The copper and iron are crucial for maintaining proper blood function and energy metabolism. Furthermore, previous reports indicate that antioxidant trace elements, such as copper and selenium, can alter the polyunsaturated-to-saturated fatty acid ratio by mitigating lipotoxicity and oxidative stress (Gouaref et al., 2024). The differences in trace element content between Yuzhong pigeons and European meat pigeons highlight the importance of breed-specific factors in determining the nutritional quality of pigeon meat. Genetic predispositions, dietary inputs, and environmental conditions all contribute to the trace element composition of the breast muscle. Yuzhong pigeons, with their higher levels of selenium, copper, and iron, may be a particularly valuable breed for consumers seeking meat that offers enhanced nutritional benefits. Understanding these variations in trace element content can inform breeding programs, dietary strategies, and nutritional recommendations aimed at maximizing the health benefits of pigeon meat. Further research is warranted to explore the underlying genetic and environmental factors that contribute to these differences, as well as the potential implications for human nutrition and food science.

## 5 Conclusion

This study analyzed and compared the nutritional composition of breast muscle in 28-day-old Yuzhong pigeons and European meat pigeons. The results indicate that Yuzhong pigeons have higher levels of monounsaturated fatty acids, umami amino acids, and essential trace elements, while European meat pigeons are richer in polyunsaturated fatty acids. Monounsaturated fatty acids, known for their cardiovascular benefits, combined with the umami amino acids in Yuzhong pigeons, enhance their appeal as a flavorful and heart-healthy meat option. Meanwhile, the polyunsaturated fatty acids in European meat pigeons, particularly omega-3 and omega-6 fatty acids, offer additional health benefits, including anti-inflammatory and cardiovascular support. These findings provide valuable baseline data and a scientific foundation for the further development and utilization of pigeon breed resources, as well as for breeding new pigeon varieties in China. Additionally, this research offers useful insights for guiding consumer choices regarding poultry meat selection based on nutritional content, making it particularly relevant for individuals seeking healthier protein options in their diet.

## Funding

This study was supported by Program for Major Scientific and Technological Special Project of Henan Province (No.221100110200).

## CRediT authorship contribution statement

**Pengkun Yang:** Writing – original draft, Visualization, Methodology, Data curation. **Xinghui Song:** Validation, Methodology, Data curation. **Wenyi Wu:** Software, Validation. **Liheng Zhang:** Methodology, Investigation. **Zhanbing Han:** Supervision. **Xinlei Wang:** Software. **Runzhi Wang:** Resources. **Mingjun Yang:** Resources. **Zhen Zhang:** Writing – review & editing, Funding acquisition, Conceptualization.

## Declaration of competing interest

The authors declare that they have no known competing financial interests or personal relationships that could have appeared to influence the work reported in this paper.

## Data Availability

No data was used for the research described in the article.
